# Year Old Eggs

**Published:** 1871-05

**Authors:** 


### YEAR OLD EGGS.

TAKE three gallons of pure water, add one pint of unslacked lime and half a pint of common salt, filling the vessel half full; let the eggs down in such a way as not to crack the shell. If this is well done, the eggs will keep a year, and at the end of that time will be almost as good as when first put in the mixture; there should be two or three inches of the liquid above the highest layer of eggs, so as to keep out the air. It is the passage of air through the shell, which is full of pores, that destroys the egg; the lime and salt fill up these pores very effectually.

